# Construction of an Evidence Ecosystem‐Based Postoperative Pain Management Programme for Paediatric Patients

**DOI:** 10.1002/nop2.70167

**Published:** 2025-03-02

**Authors:** Jin‐Xia Yang, Yue Liu, Zhen Yu, Fang Zhang, Wen‐Ying Yao, Guo‐Ying Wang, Zi‐sheng Ai

**Affiliations:** ^1^ Basic Medical Science Tongji University School of Medicine Shanghai China; ^2^ Department of Nursing Children's Hospital of Soochow University Suzhou China; ^3^ Department of Orthopedics Shanghai Pudong New Area Gongli Hospital Shanghai China

## Abstract

**Aim:**

To construct an evidence ecosystem‐based postoperative pain management programme for children with postoperative pain management.

**Design:**

A mixed research design that combines qualitative and quantitative studies.

**Methods:**

According to the literature search and analysis, the postoperative pain management programme for children was constructed from three aspects: assessment of pain intensity of children, management principles and management methods, and the preliminary draft of the programme was finally constructed to include three first‐level entries and 11 second‐level entries. In January–February 2023, the first draft of the postoperative pain management programme for children was developed based on a literature review using the ecosystem of evidence theory as the research framework; in March–April 2023, the postoperative pain management programme for children was revised and finalised through two rounds of Delphi expert consultation.

**Results:**

In the second round of expert consultation, the return rate of valid questionnaires was 100%, the expert authority coefficient was 0.83, the importance scores and feasibility scores of each entry were > 3.5, the coefficients of variation were < 0.25, the Kendall's harmony coefficients of the importance scores of the entries were 0.650 (*χ* ^2^ = 273.134, *p* < 0.001) and those of the feasibility scores were 0.649 (*χ* ^2^ = 272.720, *p* < 0.001). The resulting postoperative pain management programme for the affected patients included three level 1, 11 level 2 and eight level 3 entries.

**Patient or Public Contribution:**

The postoperative pain management programme for children constructed based on the evidence ecosystem is practical and scientific, but its effectiveness in clinical practice needs to be further verified by a controlled study design.

**Objectives:**

Evidence ecosystem; paediatrics, surgery; pain; Delphi method.

## 1 Introduction

### 1.1 Background

The International Association for Pain defines pain (Pain) as being an unpleasant sensory and emotional experience that is considered to be caused by actual or possible tissue damage (Soleymanifard et al. 2014). Postoperative pain (POP) is an adaptive response to physical injury due to surgical procedures, a predictable, short‐term, self‐limiting outcome, and is the most common and most important type of acute injurious pain to be given rapid management in surgical units (Birnie et al. 2019), usually occurring in the first 24–72 h after surgery, but also can last for days or weeks. There is growing evidence that postoperative pain induces a series of negative physiological and psychological experiences in the body. Physiologically, inadequate postoperative pain management in children can lead to the activation of physiological and biochemical stress responses, resulting in impaired metabolic, endocrine, neurological, pulmonary and immune functions, and the unpleasant experience of pain or emotional state may trigger disturbances in plasma cortisol levels, blood oxygen saturation, heart rate and respiratory rate which may affect the child's sleep, lead to crying and increase the incidence of complications such as incision dehiscence and bleeding (Lan et al. 2007; Ting et al. 2022). Psychologically, pain may lead to negative emotions such as fear, anxiety and depression in the short term (Sng et al. 2017 ), as well as behavioural changes, sensory‐cognitive deficits and temperament changes in the long term. Although postoperative pain management guidelines are constantly updated, there is a large discrepancy between clinical practice and guideline evidence, and it is a difficult task to translate guidelines into applicable and measurable indicators in the clinical practice setting. The concept of Evidence Ecosystem was first proposed by MAGIC (Making GRADE the irresistible choice), which covers six aspects: generation of evidence, synthesis of evidence, dissemination of evidence to clinical practitioners, dissemination of evidence to patients, application of evidence and evaluation and improvement of practice. To promote the dynamic and continuous cycle and transformation of evidence in the process of ‘generation, integration, dissemination, application, evaluation and improvement’ (Yingfeng et al. 2019). In this study, we will follow the evidence ecosystem, search and summarise and analyse the domestic and international evidence on postoperative pain management in children, and construct a set of scientific and evidence‐based postoperative pain management programme for children on this basis, and the methodology and results are now reported as follows.

### 1.2 Purpose

To construct an evidence ecosystem‐based postoperative pain management programme for children with postoperative pain management.

## 2 Methods

### 2.1 Establishment of the Programme Building Team

A protocol construction team was set up, consisting of one director of the nursing department, one deputy chief surgeon, two chief nurses of surgery, one deputy chief surgeon of the anaesthesiology department and two master's degree students in nursing who have studied the evidence‐based nursing system. The director of the Department of Nursing was responsible for technical guidance and coordination and communication during the construction of the protocol; the deputy chief surgeon, chief nurse of the Department of Surgery and deputy chief surgeon of the Department of Anaesthesiology were responsible for the review of the first draft of the protocol and the selection of experts for the Delphi Expert Consultation and the initial communication and master's degree nursing graduate students were responsible for the development of the first draft of the protocol and the implementation of the Delphi Expert Consultation, which specifically included the literature retrieval, screening and extraction of the protocol entries and the questionnaires for the Expert Consultation were distributed, retrieved and analysed.

### 2.2 Initial Construction Programme

#### 2.2.1 Theoretical Framework

The concept of Evidence Ecosystem (Evidence Ecosystem) was first proposed by MAGIC (Making GRADE the irresistible choice) International Organisation, which covers six links: generation of evidence, synthesis of evidence, dissemination of evidence to clinical practitioners, dissemination of evidence to patients, application of evidence and evaluation and improvement of practice. To promote the dynamic and continuous cycle and transformation of evidence in the process of ‘generation, integration, dissemination, application, evaluation and improvement’.

#### 2.2.2 Literature Research

1. Search strategy. Following the PIPOST principle, P (Population): postoperative children (excluding neonates); I (Interventions): postoperative pain management in children; P (Professional): surgical nurses, surgeons, anaesthesiologists, pharmacists, etc.; O (outcomes): postoperative pain scores in children; S (Setting): Lack of evidence‐based practice experience and lack of specifications for postoperative pain management in children by surgical nursing staff in a tertiary care children's hospital; T (Type of evidence): clinical practice guidelines, evidence summaries, systematic evaluations, expert consensus
2. Retrieved keywords. English keywords: ‘child OR children OR infant OR pediatric OR newborn OR neonate OR adolescent OR teenager’ AND ‘post‐operative OR postoperative OR post surgery OR post‐surgical OR perioperative period’ AND ‘pain OR ache OR ERAS OR FST OR analgesia’ AND ‘systematic review OR meta‐analysis OR guideline OR evidence summary OR consensus’; Chinese keywords: ‘child OR children OR infants OR neonates OR adolescents’ AND ‘preoperative OR perioperative’ AND ‘pain OR analgesia OR rapid recovery OR accelerated recovery’ AND ‘systematic evaluation OR meta‐analysis OR guideline OR evidence summary OR expert consensus’
3. Evidence resource databases searched. English: EMJ, NICE, American Guidelines Network, JBI, Cochrane Library, Pubmed, American Society of Anesthesiologists, American Academy of Pediatrics, American Pain Society, Canadian Medical Association Clinical Practice Guidelines Network, Scottish Intercollegiate Guidelines Network, New Zealand Guidelines Collaborative Group Network, International Guidelines Collaborative Group Network. The four major Chinese networks: CBM, Wanfang, Wipu and China Knowledge.
4. Inclusion and exclusion criteria for evidence resources. Inclusion criteria: the topic was postoperative pain management in children; the language limit was Chinese and English; the time limit was the literature in the last 10 years (2012 to present); and the type of literature was clinical guidelines, best practices, evidence summaries, systematic evaluations and expert consensus. Exclusion criteria: content nonconformity, intervention nonconformity and original literature, grey literature.
5. General status of the included literature. Initially, 669 articles were retrieved, including 346 articles in English and 323 articles in Chinese; after reading by two researchers, 78 articles were excluded as duplicates, population nonconformity, intervention nonconformity, solution nonconformity and duplicated evidence. Eleven papers were finally included in this study, including six guidelines, three systematic evaluations and two meta‐analyses. The basic information of the included literature is shown in Table 1.
6. Literature quality evaluation criteria and process. Guidelines were evaluated for quality using the 2009 UK Appraisal of Guidelines for Research and Evaluation (AGREE II) (Siler et al. 2019); systematic evaluations and meta‐analyses were performed using the JBI 2015 version of the standard (Assessment of Multiple Systematic Reviews, AMSTAR) (Pancekauskaitė and Jankauskaitė 2018) for quality evaluation of systematic evaluation literature. The quality evaluation of the literature was done independently by two researchers who had been trained in the ‘Fudan University Evidence‐Based Continuous Quality Improvement Training Camp’, and if there was any disagreement, the two of them discussed and invited experts in evidence‐based nursing to guide and reach a consensus.

**TABLE 1:** General information about the included literature.

Included literature	Source of literature	Publishing organisation	Type of evidence	Subject of literature	Evidence grading system	Date of publication (year)
Maciej et al.	Pubmed	Paediatric Section of the Polish Society of Anaesthesiology and Intensive Therapy	Guidelines	Treatment of acute pain in children	David Sackett (1986)	2022
Ross et al.	Pubmed	Ross et al.	Systematic evaluation	Postoperative pain in children and adolescents	/	2018
Lorraine et al.	JAMA Surgery	MSHS	Guidelines	Postoperative opioid use in children and adolescents	GRADE	2022
Kathryn et al.	Pubmed	/	Systematic evaluation	Pain assessment in children and adolescents	/	2019
Fiona et al.	Pubmed	/	Meta‐analysis	Psychosocial intervention for postoperative pain in children	/	2016
Dana et al.	Pubmed	/	Guidelines	Postoperative pain management in children undergoing cardiac surgery	JBI	2022
Peter et al.	Pubmed	Netherlands Society of Anesthesiology	Guidelines	Postoperative pain management	JBI	2013
Sng et al.	JBI	Australian Centre for Evidence‐Based Health Care	Meta‐analysis	Postoperative pain management experience for children	JBI	2017
Boric et al.	Wiley	/	Systematic evaluation	Postoperative pain in children	/	2017
Chou et al.	APS	American Pain Society	Guidelines	Postoperative pain management	GRADE	2016
Maria et al.	Pubmed	ESPA	Guidelines	Postoperative pain management in children	GRADE	2018

#### 2.2.3 Formulation of the First Draft

In this study, based on evidence‐based methodology, evidence on ‘postoperative pain management in children’ was extracted from the selected literature based on the inclusion and analysis of the literature, and each piece of evidence was evaluated using the FAME principle, and finally aggregated into the best evidence. Among the members of the research team, the best evidence was discussed and analysed using the focus group method, and after the discussion, it was agreed that a postoperative pain management programme for children should be constructed from the three aspects of assessment of pain intensity, management principles and management methods. At the same time, the best evidence was summarised into an initial pool of entries corresponding to the three dimensions of pain intensity assessment, management principles and management methods. The final constructed initial draft of the programme included three level 1 entries and 11 level 2 entries. The preliminary draft programme was also transformed into the first round of the expert consultation questionnaire, which included an introduction to the purpose and significance of the study, an evaluation of the importance and feasibility of the programme entries, general information about the experts, the experts' familiarity with the field and the basis for the evaluation judgement.

### 2.3 Expert Consultation

#### 2.3.1 Preparation of Expert Consultation Questionnaire

The expert correspondence questionnaire has four parts: (1) Preamble, which introduces the background, purpose, significanceand method of this study to the correspondence experts. (2) Basic information questionnaire for the experts, including gender, age, highest education, title, position, years of experience in the specialty, familiarity with the content of the correspondence and the basis for judgement. (3) Evaluation table of indicators at all levels, each entry using Likert 5‐level scoring, from ‘strongly disagree’ to ‘strongly agree’ were assigned the value of 1, 2, 3, 4 and 5; the higher the score, that is, the higher the level of satisfaction with the content of the entry and with the opinion of the revised. The higher the score, the higher the satisfaction with the content of the entry, and a revised column with comments. Each round of expert consultation invites experts to evaluate the importance and feasibility of each entry while setting up subjective questions, including: How do you think the entry needs to be changed? Do you think there are any other entries that need to be added or deleted? Do you have any other suggestions? (4) Appendices, including the contact information of the research team and relevant references.

#### 2.3.2 Process of Implementing Expert Consultation

After the formation of the expert correspondence questionnaire, the first draft was revised and improved through two rounds of consultation with 20 experts in the fields of nursing, paediatric surgery and anaesthesiology. The criteria for inclusion of experts were as follows: (1) the work unit was a tertiary‐level hospital; (2) the work position was in the fields of nursing, paediatric surgery, anaesthesiology, etc.; (3) the education was at the undergraduate level or above and (4) the title was at the associate level or above. The questionnaires were distributed by mail, and a draft of the correspondence was requested to be returned within 2 weeks after the distribution of each round of questionnaires. After the questionnaires were returned, the quality of the questionnaires was evaluated by the degree of positivity and authority coefficient of the experts, and the degree of coordination of the experts' opinions. After the first round of expert consultation, the research team summarises, organises and analyzes the experts' opinions and forms the second round of consultation questionnaires on this basis. The expert opinions converge after the second round of expert consultation and end the consultation.

### 2.4 Statistical Methods

SPSS 20.0 software was used for data processing and statistical analysis; percentages were used for statistical description, and internal consistency analysis and content validity were used for statistical inference.

## 3 Results

### 3.1 Basic Information of Experts

In Delphi Expert Consulting, choosing the right expert is key to ensuring the quality of the study and the reliability of the results. In this study, senior experts in the fields of nursing, paediatric surgery and anaesthesiology were finally selected based on a combination of professional knowledge and experience, representativeness, authority, familiarity with the study content, motivation and participation. The number of experts consulted in a study should be moderate, generally between 10 and 50, to ensure that knowledge and experience in each field are covered, and that the discussion process is not overly complicated, and 20 experts from tertiary‐level hospitals were finally selected for this study. In two rounds of correspondence, of which 15 were aged 40 to < 50 years old, and five were aged 50 years old and above; years of experience (21.65 ± 1.22) years; two had PhDs, nine had master's degrees and nine had bachelor's degrees; seven had full‐senior titles, and 13 had vice‐senior titles; 15 were experts in paediatric nursing, two were experts in paediatric surgical care, two were experts in anaesthesiology and one was an expert in medical statistics. 1 expert.

### 3.2 Results of Expert Consultation

The reliability of each entry of the questionnaire was judged by the degree of enthusiasm of the consulted experts, the degree of authority of the experts and the coefficient of coordination of the experts’ opinions.

#### 3.2.1 The Degree of Enthusiasm and Authority of the Experts

Both rounds of expert consultation issued 20 questionnaires and recovered 20 valid questionnaires, and the recovery rate of valid questionnaires was 100%, indicating that the experts have a high degree of concern and enthusiasm for this study. In the first round of expert consultation, six experts put forward eight modifications, and one expert put forward one modification in the second round. In the two rounds of expert consultation, the coefficient of judgement (Ca) of experts was 0.84, the degree of familiarity of experts with the entries (Cs) was 0.82 and the coefficient of authority (Cr) of experts was 0.83, which was greater than 0.80, which indicated that the experts' authority level was higher.

#### 3.2.2 Degree of Coordination of Experts' Opinions

In the first round of expert consultation, the coefficient of variation of the entry importance score was 0.05–0.18 and the Kendall's harmony coefficient was 0.544 (*χ* ^2^ = 141.534, *p* < 0.001); the coefficient of variation of the feasibility score was 0.05–0.14 and the Kendall's harmony coefficient was 0.688 (*χ* ^2^ = 178.801, *p* < 0.001). In the second round of expert consultation, the coefficient of variation (CV) of the entry importance score was 0.05–0.18 and the Kendall's harmony coefficient was 0.650 (*χ* ^2^ = 273.134, *p* < 0.001); the coefficient of variation (CV) of the feasibility score was 0.05–0.18 and the Kendall's harmony coefficient was 0.649 (*χ* ^2^ = 272.720, *p* < 0.001). This indicates that there is no major disagreement among experts on this entry.

#### 3.2.3 Modification of the Entry

After two rounds of expert consultation, the questionnaire was modified according to the expert consultation opinions, and the final entries had a mean importance score of 3.45–4.95 and a mean feasibility score of 3.45–4.95. Results of the second round of expert consultation is shown in Table 2. In the first round of expert consultation, eight tertiary entries were added, and the secondary entries ‘route of administration of analgesics’ and ‘prophylactic analgesia’ were subdivided, of which ‘route of administration of analgesics’ was added to ‘oral administration’. Six entries corresponding to oral, intravenous, rectal, continuous subcutaneous, topical and alternative routes of administration were added to ‘Route of administration of analgesics’, and two entries were added to ‘Prophylactic analgesia’. In the second round of expert consultation, one secondary entry was amended to delete morphine from the list of continuous subcutaneous administration in consideration of the special characteristics of medication for children, and it was proposed that the entry be amended to read: ‘Continuous subcutaneous administration: continuous subcutaneous supply of analgesic is also recommended for postoperative analgesia in children’.

**TABLE 2:** Results of the second round of expert consultation on the postoperative pain management programme for children.

Item	Significance				Feasibility			
	Mean	95% CI	Standard deviation	Coefficient of variation	Mean	95% CI	Standard deviation	Coefficient of variation
1. Basic principles of assessment	4.95	(4.85–5.05)	0.224	0.05	4.95	(4.85–5.05)	0.224	0.05
1.1. Perform regular pain assessments in children and immediately when the child develops pain	4.90	(4.76–5.04)	0.308	0.06	4.60	(4.36–4.83)	0.503	0.11
1.2. Select the appropriate pain assessment tool according to the child's age and cognitive level	4.95	(4.84–5.05)	0.224	0.05	4.95	(4.85–5.05)	0.224	0.05
2. Basic principles of management	4.95	(4.85–5.05)	0.224	0.05	4.95	(4.85–5.05)	0.224	0.05
2.1. Choice of analgesic method: consider the child's age, previous pain experience, type of surgery, expected pain intensity and duration	4.95	(4.85–5.05)	0.224	0.05	4.95	(4.85–5.05)	0.224	0.05
2.2. Frequency of analgesic administration: based on the child's age and age‐related pharmacology of the drug, choose to administer the analgesic by continuous infusion or at equal intervals, thus maintaining a constant blood concentration of the analgesic to provide effective analgesia	4.90	(4.76–5.04)	0.308	0.06	4.60	(4.36–4.83)	0.503	0.11
2.3. Routes of analgesic administration	4.95	(4.85–5.05)	0.224	0.05	4.95	(4.85–5.05)	0.224	0.05
2.3.1. Oral administration: the usual route for children	4.90	(4.76–5.04)	0.308	0.06	4.55	(4.31–4.79)	0.510	0.11
2.3.2. Intravenous administration: when there is an impairment in the child's ability to swallow, the intravenous route is considered, with administration of repeated single‐dose analgesics at equal intervals, or continuous infusion	4.70	(4.43–4.97)	0.571	0.12	4.55	(4.27–4.83)	0.605	0.13
2.3.3. Rectal administration: in younger children, if oral or intravenous analgesics are not available, rectal administration is an option (this route should be avoided in immunosuppressed children, with the risk of perianal abscesses)	4.55	(4.19–4.91)	0.759	0.17	4.55	(4.19–4.91)	0.759	0.17
2.3.4. Continuous subcutaneous administration: a continuous subcutaneous supply of analgesics is also recommended in children with postoperative analgesia	3.45	(3.21–3.69)	0.510	0.15	3.45	(3.21–3.69)	0.510	0.15
2.3.5. Topical medications: use transdermal patches	3.70	(3.39–4.01)	0.657	0.18	3.70	(3.39–4.00)	0.657	0.18
2.3.6. Alternative routes of administration: these include nasal or transmucosal supply (oral, sublingual), which may be used when intravenous administration is not possible or acceptable. The nasal route may be used for the treatment of potent opioids such as fentanyl, sufentanil and ketamine	3.75	(3.49–4.01)	0.550	0.15	3.75	(3.49–4.01)	0.550	0.15
2.4. Prophylactic analgesia	4.95	(4.85–5.05)	0.224	0.05	4.95	(4.85–5.05)	0.224	0.05
2.4.1. Severe pain in children should be prevented by providing prophylactic analgesia that may lead to a reduction in the intensity of postoperative pain, thereby reducing the need for analgesics	4.95	(4.85–5.05)	0.224	0.05	4.90	(4.76–5.04)	0.308	0.06
2.4.2. Preferably, the surgical wound should be injected with local anaesthesia prior to surgery or at least until the surgery is completed	4.80	(4.56–5.04)	0.523	0.11	4.70	(4.43–4.97)	0.571	0.12
2.5. Nonpharmacological analgesia: Nonpharmacological methods (distraction methods) such as fairy tales, movies or toys should be considered	4.95	(4.85–5.05)	0.224	0.05	4.90	(4.76–5.04)	0.308	0.06
3. Management methods	4.95	(4.84–5.05)	0.224	0.05	4.95	(4.85–5.05)	0.224	0.05
3.1. Multimodal analgesia: Pain management in children should be based on the principle of multimodal analgesia	4.95	(4.84–5.05)	0.224	0.05	4.95	(4.85–5.05)	0.224	0.05
3.2. Pharmacologic treatment of pain: including opioids, nonopioid analgesics, acetaminophen, temizole and nonsteroidal anti‐inflammatory drugs	4.55	(4.27–4.83)	0.605	0.13	3.95	(3.85–4.05)	0.224	0.06
3.3. Combined analgesics: gabapentin analogs, alpha‐2 adrenergic receptor agonists, ketamine, corticosteroids and magnesium sulfate may be used as combined analgesics for pain management in children	4.00	(3.66–4.34)	0.725	0.18	3.80	(3.56–4.04)	0.523	0.14
3.4. Co‐analgesics in regional anaesthesia for children: alpha2‐agonists	3.70	(3.48–3.92)	0.470	0.13	3.70	(3.48–3.92)	0.470	0.13

#### 3.2.4 Reliability

The final postoperative pain management programme for children included three dimensions and 22 entries, with a total Cronbach's ɑ for the questionnaire of 0.857, and Cronbach's α for the three dimensions of basic principles of assessment, basic principles of management and management methods of 0.801, 0.700 and 0.839 respectively. Cronbach's α for each entry was 0.859 to 0.883.

#### 3.2.5 Content Validity

The I‐CVI of the programme ranged from 0.80 to 0.95, and the S‐CVI was 0.90, indicating that the programme has good content validity.

#### 3.2.6 Structural Validity

**FIGURE 1:**
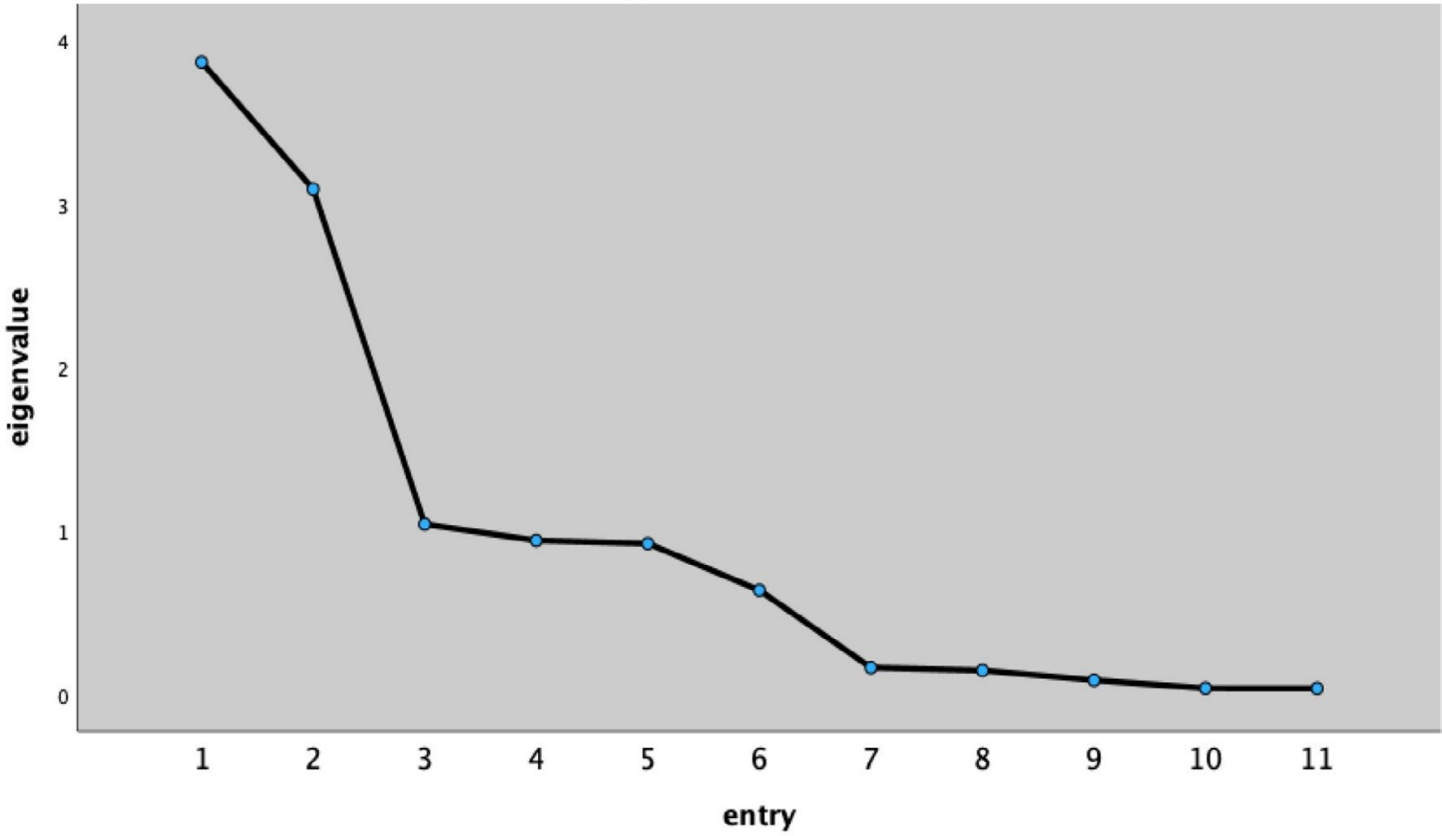
Gravel chart.

Structural validity was analysed using EFA. The postoperative pain management programme for children had a KMO value of 0.741 for the 11 secondary entries and a Bartlett's spherical statistic value of 1018.877 (df = 55, *p* < 0.001), making it suitable for factor analysis. Factor analysis was performed using principal component analysis with eigenvalues > 1 and orthogonal rotation by the maximum variance method, and seven common factors were extracted with a cumulative contribution of 72.744%. Moreover, the factor loadings of each entry on their respective dimensions were > 0.5, which is shown in Table 3, and the gravel plot formed gravel after the third factor is shown in Figure 1, indicating that the extracted factors were acceptable, and the validity of the questionnaire was good. Structural validity.

**TABLE 3:** Factor analysis results of the postoperative pain management programme for children.

Item	Factor		
	1	2	3
1.1. Perform regular pain assessments in children and immediately when the child develops pain	−0.316	0.439	0.508*
1.2. Select the appropriate pain assessment tool according to the child's age and cognitive level	−0.274	0.000	0.580*
2.1. Choice of analgesic method: consider the child's age, previous pain experience, type of surgery, expected pain intensity and duration	0.936*	0.285	0.051
2.2. Frequency of analgesic administration: based on the child's age and age‐related pharmacology of the drug, choose to administer the analgesic by continuous infusion or at equal intervals, thus maintaining a constant blood concentration of the analgesic to provide effective analgesia	0.929*	0.246	0.058
2.3. Routes of analgesic administration	0.895*	0.309	0.052
2.4. Prophylactic analgesia	0.904*	0.251	0.128
* 2.5. Nonpharmacological analgesia: Nonpharmacological methods (distraction methods) such as fairy tales, movies or toys should be considered	0.010	0.291	−0.779
3.1. Multimodal analgesia: Pain management in children should be based on the principle of multimodal analgesia	−0.252	0.842*	0.061
3.2. Pharmacologic treatment of pain: including opioids, nonopioid analgesics, acetaminophen, temizole and nonsteroidal anti‐inflammatory drugs	−0.281	0.857*	0.008
3.3. Combined analgesics: gabapentin analogs, alpha‐2 adrenergic receptor agonists, ketamine, corticosteroids and magnesium sulfate may be used as combined analgesics for pain management in children	−0.396	0.658*	−0.008
3.4. Co‐analgesics in regional anaesthesia for children: alpha2‐agonists	−0.187	0.798*	−0.103
Eigenvalue	3.867	3.091	1.044
Variance contribution	35.153	28.103	9.487
Cumulative variance contribution	35.153	63.257	72.744

*Note:* Bolded values represent factor loadings of the entry are more than 0.5.

* Factor loading of the entry is less than 0.5.

## 4 Discussion

### 4.1 The Postoperative Pain Management Programme for Children Has Good Scientific Validity and Reliability

The programme was constructed under the theoretical framework of evidence ecosystem, with sufficient theoretical basis; the construction method followed the guiding principle of evidence‐based nursing practice, and the programme was constructed from the three aspects of assessment of children's pain intensity, management principles and management methods through the summary of the best evidence from six guidelines, three systematic evaluations and two meta‐analyses, with reliable methods. In Delphi expert consultation, Kendall's harmony coefficients are commonly used in evaluator consistency analyses, with values ranging from 0 to 1. The closer it is to 1, the higher the consistency between rankings; the closer it is to 0, the lower the consistency between rankings. The coefficient of variation can be used to compare the degree of dispersion of different data sets. Higher coefficients of variation indicate more dispersed data; lower coefficients of variation indicate more concentrated data. The results of the study showed that in the two rounds of expert consultation, the recovery rate of valid questionnaires was 100%, and the expert authority coefficient was 0.83; the mean importance scores of the final entries ranged from 3.45 to 4.95, and the Kendall's harmony coefficient was 0.650 (*χ* ^2^ = 273.134, *p* < 0.001); the mean feasibility scores ranged from 3.45 to 4.95, and the Kendall's harmony coefficient was 0.649 (*χ* ^2^ = 272.720, *p* < 0.001); the coefficients of variation for each entry were less than 0.25, indicating that the expert opinions had converged. However, we also observed that in the section ‘Routes of analgesic administration’, the coefficients of variation for the entries on rectal administration, continuous subcutaneous administration, parenteral administration and alternative routes of administration were relatively high compared to the other entries. Inspection of the original rating records revealed that the importance and feasibility of this series of entries were mainly rated higher by experts in the field of anaesthesia and lower by experts in the field of nursing. Analysing the reasons, it may be that anaesthetists use subcutaneous and oral‐nasal inhalation routes of analgesic administration more frequently in clinical practice, while nurses use oral administration and intravenous routes more frequently, which leads to differences in the judgements of importance and feasibility of the routes of analgesic administration made by experts in various fields. This also suggests that multidisciplinary cooperation needs to be further strengthened in the future management of postoperative pain in children, thus facilitating the application and enhancement of multimodal analgesic modalities. In addition, this programme covers pain assessment and management, and the specific content involves comprehensive assessment of postoperative pain, multimodal pain management and nonpharmacological interventions, which is more comprehensive, indicating that the postoperative pain management programme for children has good scientific validity and reliability.

### 4.2 Analysis of the Significance and Content of the Construction of the Postoperative Pain Management Programme for Children

#### 4.2.1 Children's Pain Is Unique and Challenging to Assess and Manage

Pain assessment tools for children are highly specific and cannot be homogenised for different ages. Cognitive developmental factors can influence children's understanding of pain and their ability to describe pain effectively, and good measures ‘should accurately reflect an individual's subjective experience’. Pain perception is fully developed around 25 weeks of gestation, whereas endogenous downstream inhibitory pain pathways do not develop until mid‐infancy. All of these increase the inflammatory response to noxious stimuli in children compared to adults, resulting in more pain‐related physiologic changes. Currently, the use of age‐specific pain rating scales to objectively assess pain levels for children remains essential for providing effective pain management (Siler et al. 2019; Pancekauskaitė and Jankauskaitė 2018). Specifically, these include: neonatal period and infancy (0–3 years): in neonates, infants and toddlers, where it is often difficult to assess pain as much as crying, a common symptom that occurs in other nonpainful situations, pain is often assessed by behavioural and physiological parameters using the Preterm Infant Pain Profile Scale, the CRIES Post‐Operative Pain Scale and the FLACC Scale; preschoolers and preschool‐aged children (3–8 years): a simple self‐assessment scale with different facial expressions is usually used to describe the level of pain; children older than 8 years: their pain can be easily described using the Visual Analog Scale (VAS) and the Verbal Numeric Pain Rating Scale (Mehrotra 2019; RNAO 2024; Neonatologists Branch of Chinese Physicians Association, Editorial Committee of Chinese Journal of Contemporary Pediatrics 2020; Wilson 2019). The ‘basic principles of assessment’ dimension of this protocol emphasises the need to select the appropriate pain assessment tool according to the age and cognitive level of the child, which is consistent with the unique nature of pain in children.

#### 4.2.2 Postoperative Pain Has a Complex Mechanism and Seriously Jeopardises the Life and Health of Children

Postoperative pain is a kind of acute pain caused by surgical trauma, including the inflammatory response and the initiation of obstruction in afferent neurons, which transmit sensory signals to the brain. Postoperative pain induces a series of neuroendocrine stress responses in the body, such as the abnormal release of nociceptive inflammatory mediators, which can lead to a decrease in immunoglobulins and impede wound healing in children, thus affecting postoperative recovery (Hobson et al. 2015). Pain has a significant impact on the physiological and psychological health of neonates, infants and children. In humans, the process of maturation of the central nervous system takes a long time, and persistent pain stimulation early in life is a factor that may impair the maturation process of the central nervous system and significantly modulate pain behaviour later in life (Cettler et al. 2022). There is growing evidence that ineffective treatment of postoperative pain is associated with delayed wound healing and with future pain perception, pain behaviours and the onset of chronic pain, as studies have shown that severe pain lasting more than 2 weeks postoperatively is a risk factor for the development of chronic pain within a year (Cettler et al. 2022; Boric et al. 2017; Xiuli et al. 2014; Yan et al. 2008; Zhou et al. 2022; Shen and Huang 2021). It is certain that poor postoperative pain management leads to the development of complications and prolonged recovery time (Boric et al. 2017).

## 5 Conclusion

This study constructed a postoperative pain management programme for children based on an evidence‐based ecosystem, and the research process was theoretically well‐founded, methodologically reliable and the constructed programme was comprehensive, with good scientific merit and practicality, which is committed to improving children's comfort, reducing complications and promoting children's postoperative recovery through effective pain management. However, the effectiveness of the programme in clinical practice needs to be further verified by a controlled study design.

## Author Contributions

Jin‐xia Yang, Yue Liu contributed equally. Jin‐Xia Yang contributed to the study design. Wen‐Ying Yao, Fang Zhang, Zhen Yu and Guo‐Ying Wang were responsible for data collection. Jin‐Xia Yang, Yue Liu and Zi‐sheng Ai carried out data analysis. Jin‐Xia Yang prepared the manuscript and all authors gave the final approval.

## Ethics Statement

The study was approved by the hospital ethics committee (ethics code: 2021CS063).

## Conflicts of Interest

The authors declare no conflicts of interest.

## Data Availability

The data that support the findings of this study are available from the corresponding author upon reasonable request.
